# Special Invertebrate Models and Integrative Medical Applications: Regulations, Mechanisms, and Therapies

**DOI:** 10.1155/2014/843961

**Published:** 2014-05-06

**Authors:** Chih-Yang Huang, Edwin L. Cooper, Catherine Fang-Yeu Poh, Wei-Wen Kuo, Tung-Sheng Chen, Ronald Sherman

**Affiliations:** ^1^Graduate Institute of Basic Medical Science, China Medical University, Taichung 40402, Taiwan; ^2^Department of Biotechnology, Asia University, Taichung 41354, Taiwan; ^3^Laboratory of Comparative Neuroimmunology, Department of Neurobiology, David Geffen School of Medicine at UCLA, University of California, Los Angeles, CA 90095-1763, USA; ^4^Faculty of Dentistry, The University of British Columbia, 2151 Wesbrook Mall, Vancouver, BC, Canada V6T 1Z3; ^5^Department of Biological Science and Technology, China Medical University, Taichung 40402, Taiwan; ^6^Bio Therapeutics, Education & Research Foundation, Irvine, CA 92617, USA

This special issue of eCAM has brought together several unique investigators interested in invertebrate animal models. Investigators would benefit by examining advances and applications of findings derived from useful terrestrial and marine invertebrate animal models. Results derived from well-defined evidence-based approaches are usually relevant to humans; the advantages are numerous, inexpensive, and noncontroversial and are not subject to rigid, ethical, or moral guidelines.

In the historical context, these animals have been crucial in many cultures before the advent of industrial medicines thus providing the only source of aid. Now, there is a move to return to or at least to include these practices lest they disappear since, in certain cultures, they represent the sole source of useful health remedies. However, evidence-based medicine emphasizes the importance of subjecting animal products to rigorous analysis. Results are then standardized and serve to educate populations including those that use exclusively western practices.

eCAM stands ready to accept the importance and contributions of bees as valuable sources of beneficial activities (pollination) and products (honey and propolis) that for centuries have enhanced human lives as food and medicine. Others include corals, millipedes, maggots, and earthworms. Corals are marine invertebrates that live in compact colonies. Humans have learned that they are useful in the manufacture of jewelry and chemical compounds that are useful against cancer, AIDS, and pain and serve as skeletons during bone grafting. Millipedes are arthropods (the same group as shrimps, crabs, butterflies, and bees) which in some cultures are used during pregnancy and as cures for fever, wounds, earaches, and hemorrhoids and food among the Bobo people of Burkina Faso when crushed; sometimes they are boiled and eaten in tomato sauce.

Since the time of Napoleon and even centuries earlier, maggots have been used especially in patients with severe intractable wounds caused by nerve degeneration that results from diabetes. In fact, leeches and maggots are now valuable components of the emerging field of biotherapy, the therapeutic use of living creatures to clean gangrenous tissue often found in ulcers, burns, and postoperative infections. Earthworms are experiencing growing popularity due to their many applications including sources of high protein as food and the production of an anticlotting agent known as lumbrokinase that dissolves blood clots in patients. Aside from observations and usefulness by the layman, certain techniques are valuable as molecular biologists strive to understand the minute structure of molecules in order to improve therapies. Let us not forget the contributions of earthworms as tillers of the soil which enhance agricultural output. Earthworms keep the soil aerated by their constant churning which makes for improved yield of plants regardless of the proposed use.

*Chih-Yang Huang* *Chih-Yang Huang* *Edwin L. Cooper* *Edwin L. Cooper* *Catherine Fang-Yeu Poh* *Catherine Fang-Yeu Poh* *Wei-Wen Kuo* *Wei-Wen Kuo* *Tung-Sheng Chen* *Tung-Sheng Chen* *Ronald Sherman* *Ronald Sherman*

